# Etiology of Acute Diarrheal Disease and Antimicrobial Susceptibility Pattern in Children Younger Than 5 Years Old in Nepal

**DOI:** 10.4269/ajtmh.21-1219

**Published:** 2022-12-05

**Authors:** Sanjaya K. Shrestha, Jasmin Shrestha, Carl J. Mason, Siriporn Sornsakrin, Jyoti Ratna Dhakhwa, Bhola Ram Shrestha, Bina Sakha, Jid Chani Rana, Apichai Srijan, Oralak Serichantalergs, Orntipa Sethabutr, Samandra Demons, Ladaporn Bodhidatta

**Affiliations:** ^1^ Walter Reed/AFRIMS Research Unit Nepal (WARUN), Kathmandu, Nepal;; ^2^ Centre for International Health, University of Bergen, Bergen, Norway;; ^3^ Department of Bacterial and Parasitic Diseases, Armed Forces Research Institute of Medical Sciences, Bangkok, Thailand;; ^4^ Kanti Children’s Hospital, Kathmandu, Nepal;; ^5^ Shree Mahendra Adarsa Chikitsalaya, Bharatpur Hospital, Bharatpur, Nepal

## Abstract

Diarrhea is a common cause of morbidity and mortality among children younger than 5 years in developing countries. Children from 3 to 60 months of age were recruited from two hospitals in Nepal— Bharatpur Hospital, Bharatpur, and Kanti Children’s Hospital, Kathmandu—in 2006 to 2009. Stool specimens collected from 1,200 children with acute diarrhea (cases) and 1,200 children without diarrhea (control subjects) were examined for a broad range of enteropathogens by standard microbiology, including microscopy, enzyme immunoassay for viral pathogens (adenovirus, astrovirus, and rotavirus) and protozoa (*Giardia*, *Cryptosporidium*, and *Entamoeba histolytica*), as well as by using reverse transcription real-time polymerase for norovirus. Antimicrobial susceptibility testing was performed using the disk diffusion method. Overall, rotavirus (22% versus 2%), norovirus (13% versus 7%), adenovirus (3% versus 0%), *Shigella* (6% versus 1%), enterotoxigenic *Escherichia coli* (8% versus 4%), *Vibrio* (7% versus 0%), and *Aeromonas* (9% versus 3%) were identified significantly more frequently in cases than control subjects. *Campylobacter*, *Plesiomonas*, *Salmonella*, and diarrheagenic *E. coli* (enteropathogenic, enteroinvasive, enteroaggregative) were identified in similar proportions in diarrheal and non-diarrheal stools. *Campylobacter* was resistant to second-generation quinolone drugs (ciprofloxacin and norfloxacin), whereas *Vibrio* and *Shigella* were resistant to nalidixic acid and trimethoprim/sulfamethoxazole. This study documents the important role of rotavirus and norovirus in acute diarrhea in children younger than 5 years, followed by the bacteria *Shigella*, enterotoxigenic *E. coli*, *Vibrio cholera*, and *Aeromonas*. Data on the prevalence and epidemiology of enteropathogens identify potential pathogens for public health interventions, whereas pathogen antibiotic resistance pattern data may provide guidance on choice of therapy in clinical settings.

## INTRODUCTION

Infectious diarrhea is a major cause of death in children younger than 5 years, is responsible for an estimated mortality of 525,000 children globally annually, and is associated with malnutrition in children.^1^ Etiological agents for acute infectious diarrhea include a variety of viruses, bacteria, and parasites that differ by geographic area, seasonality, population hygiene level, vaccine coverage, and age group.^2^^,^^3^ Human rotavirus is reported as a primary cause of acute diarrhea in children worldwide. *Shigella*, *Vibrio* spp., and enterotoxigenic *Escherichia coli* (ETEC) are also known pathogenic bacteria commonly causing acute diarrhea. Moreover, other pathogens such as enteroaggregative *E. coli* (EAEC), *Campylobacter*, *Aeromonas*, and *Plesiomonas* have also been associated with diarrheal disease.^4^^,^^5^ In addition, norovirus and astrovirus are also reported as diarrheal pathogens, but have been examined less often.^2^^,^^6^^,^^7^

In Nepal, according to the Ministry of Health and Population,^8^ diarrhea continues to be a major cause of childhood morbidity and mortality. In 2006, during the initiation of our study, the National Demographic and Health Survey data for children younger than 5 years showed 12% noted diarrheal episodes in that particular year, and 5% of child mortality was from diarrhea.^8^ Because of limited laboratory capabilities to detect multiple pathogens by different laboratory methods, comprehensive data on etiology of diarrhea in Nepal are not widely available. Therefore, knowledge on the different etiologies, epidemiological characteristics, and distribution of pathogens in Nepali populations may help to provide guidelines for effective intervention measures and appropriate control strategies for diarrheal disease in Nepal.

In our study we determined the prevalence of bacterial, viral, and protozoal enteropathogens in diarrhea and non-diarrhea stool samples of children younger than 5 years presenting to two hospitals located in different geographic locations in Nepal. In addition, we report the antimicrobial resistance pattern of enteric bacteria isolated in this study.

## MATERIALS AND METHODS

### Study population.

A surveillance study was conducted in children 3 months to 5 years of age with and without diarrhea in two study sites in Nepal—Bharatpur Hospital, Bharatpur, and Kanti Children’s Hospital, Kathmandu—from December 2006 through February 2009.

Bharatpur Hospital is a government district hospital located in the Bharatpur municipality of the Chitwan District. The Bharatpur municipality has a tropical climate and is located at an altitude of 208 m. It is situated 146 km southwest of Kathmandu, and has 36,939 households and a population of 143,836 (2011 national census^8^). Patients presenting to Bharatpur Hospital are mainly from the Bharatpur municipality and surrounding rural agricultural area, where the major poultry industry of Nepal is also situated.

Kanti Children’s Hospital is located in the Kathmandu District. Kathmandu is the capital of Nepal, has a high urban population density, has a temperate climate, and stands 1,400 m above sea level. The 2011 national census^8^ reported the Kathmandu District has 436,344 households and a population of 1,744,240. Kanti Children’s Hospital is also a national children’s hospital that receives patients from different parts of the country for tertiary care.

Children 3 months to 5 years seen or admitted to Bharatpur Hospital and Kanti Children’s Hospital with acute diarrhea of no more than 72 hours’ duration were enrolled as cases. Children from the same hospital seen or admitted for other causes with no history of diarrhea in the past 2 weeks were included as control subjects. Study enrollment was designed to include approximately 50 cases and 50 control subjects every 2 months for 2 years to ensure complete surveillance throughout the yearly cycles. One stool sample and brief demographic and clinical data, including antibiotic use during the past 2 weeks, were collected from each subject.

The study was approved by the Nepal Health Research Council (Kathmandu, Nepal) and the Human Subject Protection Branch, Walter Reed Army Institute of Research (Silver Springs, MD). Signed informed consent was obtained from parents or guardians of all subjects.

### Laboratory procedures.

Stool examination for white blood cells/red blood cells, ova, and parasites including *Cyclospora* was performed on a fresh stool specimen by direct stool microscopy at the two hospitals. Four swabs taken from the original stool sample were placed in modified Cary-Blair transport media. Stool was also saved in polyvinyl alcohol/formalin, and three aliquots of stool (∼1 g each) were cryopreserved and stored at –80°C at the Water Reed Armed Forces Research Institute of Medical Sciences (AFRIMS) Research Unit Nepal.

Stool from both sites was inoculated onto primary plating media and enrichment media for detection of enteric bacteria as described previously.^9^ Up to five lactose-fermenting and five non-lactose-fermenting *E. coli* colonies were isolated from each sample. Culture for *Campylobacter* was done using a Millipore filtration technique before and after enrichment, and samples were incubated under microaerophilic conditions at 37°C.^9^ Identification of bacterial enteropathogens *Shigella*,* Salmonella*,* Vibrio* spp.,* Aeromonas* spp., *Plesiomonas shigelloides*, *Arcobacter*, and *Campylobacter* spp. was performed by standard microbiological technique and biochemical testing.

Bacterial isolates were saved in a nutrient agar slant tubes and sent to AFRIMS in Thailand for confirmation, characterization, serotyping, and antimicrobial susceptibility testing. *Escherichia coli* colonies were tested by DNA hybridization with specific digoxigenin-labeled polynucleotide probes for detection of diarrheagenic *E. coli*, ETEC, EAEC, enteroinvasive *E. coli*, enteropathogenic *E. coli* (EPEC), and Shigella-like toxin-producing *E. coli*.^9^ Isolates of *Shigella*, *Salmonella*, *Campylobacter*, *Vibrio*,* Aeromonas*,* Plesiomonas*, and diarrheagenic *E. coli* (ETEC, EAEC, EPEC) were tested for antimicrobial susceptibility to azithromycin, nalidixic acid, ciprofloxacin, ampicillin, amoxicillin/clavulanic acid, trimethoprim/sulfamethoxazole, tetracycline, and cefixime using the disk diffusion method.^10^^–^^13^ These antibiotics are representative of classes of antibiotics available in Nepal.

Common intestinal parasites *Giardia lamblia* and *Cryptosporidium* were also identified at AFRIMS using a commercial ELISA kit (ProSpecT; Remel, Lenexa, KS) on stool samples. Testing for rotavirus, adenovirus, and astrovirus was performed using a commercial ELISA (RIDASCREEN; R-Biopharm, Damstadt, Germany), and norovirus was tested by reverse transcription real-time polymerase chain reaction.^14^

### Statistical analysis.

Continuous data (age) was compared using a two-tailed independent *t*-test. Proportional data, including demographic and clinical data, and number of pathogens identified in cases with diarrhea and control subjects without diarrhea were compared using two-tailed χ^2^ tests (Fisher’s exact tests for any comparisons, with *n* < 5) using SPSS (version 24; SPSS Inc., Chicago, IL). A *P* value < 0.05 was considered statistically significant. Note that multivariate analyses and categorical regression models controlling for site, age, age category, gender, or inpatient status did not alter the strength of associations appreciably and are not presented.

## RESULTS

Bharatpur Hospital and Kanti Children’s Hospital enrolled 1,200 children at each hospital, 600 children with diarrhea (cases) and 600 children without diarrhea for the past 2 weeks (control subjects). The mean age at enrollment of control subjects (22.4 months) was slightly but significantly older than cases (18.5 months). At both hospitals, children younger than 12 months were the predominant age group. Cases and control subjects were comparable for gender. At Bharatpur Hospital, most cases and control subjects were enrolled as inpatients (89% and 93%, respectively; *P* < 0.001), whereas at Kanti Children’s Hospital, 58% of cases and 39% of control subjects were enrolled as inpatients (*P* < 0.001) (Table 1). Reported prior antibiotic use was significantly more common among control subjects than cases at both sites, especially for Bharatpur Hospital (*P* < 0.001) (Table 1).

**Table 1:** Demographic characteristics of children with diarrhea and control subjects without diarrhea Bharatpur Hospital, Bharatpur, Nepal, and Kanti Children’s Hospital, Kathmandu, Nepal, enrolled in the diarrheal disease surveillance project from 2002 to 2004

Characteristic	Bharatpur Hospital			Kanti Children’s Hospital			Combined		
	Case (*n =* 600)	Control (*n =* 600)	*P* value	Case (*n =* 600)	Control (*n =* 600)	*P* value	Case (*n =* 1,200)	Control (*n =* 1,200)	*P* value
Age, months; mean ± SD	17.4 ± 13.8	21.9 ± 15.8	< 0.001*	19.7 ± 15.2	22.9 ± 16.9	< 0.001*	18.2 ± 1.4	22.4 ± 16.3	< 0.001*
Age group, year; *n *(%)									
< 1	259 (43)	194 (32)	–	256 (43)	217 (36)	–	515 (43)	411 (34)	< 0.001*
1–2	207 (35)	168 (28)	< 0.001*	167 (28)	142 (24)	< 0.001*	374 (32)	310 (26)	
2–5	134 (22)	238 (40)		177 (30)	241 (40)		311 (26)	479 (40)	
Male	388 (65)	390 (65)	NS†	365 (61)	375 (63)	NS†	753 (63)	765 (64)	NS†
Inpatient	531 (89)	560 (93)	< 0.005†	348 (58)	231 (39)	< 0.001†	879 (74)	791 (66)	< 0.001†
Prior antibiotics	20 (3)	466 (78)	< 0.001†	88 (15)	234 (39)	< 0.001†	108 (9)	700 (58)	< 0.001†

NS = not significant.

* Two tailed *t*-test for mean age comparison.

† Two tailed χ^2^ without continuity correction for other comparisons.

Despite the multiple laboratory tests conducted during our study, no enteric pathogens were identified in the stool samples of 31% of cases and 50% of control subjects; multiple pathogens were found in 38% of cases and 29% of control subjects (Table 2). When analyzed for bacterial pathogens, children not treated with antibiotics (1,092 cases and 500 control subjects) showed bacterial infection in 41.7% cases and 40.8% in control subjects. Rotavirus and norovirus were the two most common pathogens associated with diarrhea, identified in 21% and 13% of stool samples, respectively, and detected significantly less often in stool samples of control subjects (2% for rotavirus and 7% for norovirus). Other pathogens such as *Shigella*, ETEC, *Aeromonas*, and *Vibrio* as well as adenovirus were also identified significantly more frequently in stool samples from children with diarrhea than in those without (control subjects). *Campylobacter*,* Salmonella*, EPEC, EAEC, *Plesiomonas*, *Cryptosporidium*, and *Cyclospora* were identified in almost equal numbers in stool samples from cases and control subjects. In contrast, *Giardia* was detected significantly more often in stool samples from control subjects than cases (*P* < 0.001) (Table 2).

**Table 2:** Major enteropathogens identified in stool samples from children age 3 months to 5 years with diarrhea and control subjects without diarrhea at Bharatpur Hospital and Kanti Children’s Hospital

Enteropathogen	Bharatpur Hospital			Kanti Children’s Hospital			Combined		
	Case (*n =* 600), *n* (%)	Control (*n =* 600), *n* (%)	*P* value*	Case (*n =* 600), *n *(%)	Control (*n =* 600), *n* (%)	*P* value*	Case (*n =* 1,200), *n* (%)	Control (*n =* 1,200), *n* (%)	*P* value*
*Campylobacter*	11 (2)	29 (5)	0.004	86 (14)	78 (13)	NS	97 (8)	107 (9)	NS
*Salmonella*	12 (2)	14 (2)	NS	25 (4)	21 (4)	NS	37 (3)	35 (3)	NS
*Shigella*	24 (4)	1 (0.2)	< 0.001	48 (8)	10 (2)	< 0.001	72 (6)	11 (1)	< 0.001
*Escherichia coli*									
ETEC	19/354 (5)	14/406 (3)	NS	46/418 (11)	24/464 (5)	0.001	65/772 (8)	38/870 (4)	0.001
EPEC	22/401 (5)	36/450 (8)	NS	34/461 (7)	47/518 (9)	NS	56/862 (6)	83/968 (9)	NS
EAEC	70/401 (17)	78/450 (17)	NS	86/461 (19)	91/518 (18)	NS	156/862 (18)	169/968 (17)	NS
EIEC	0/401 (0)	3/450 (1)	NS	6/461 (1)	3/518 (0.6)	NS	6/862 (1)	6/968 (1)	NS
*Aeromonas*	36 (6)	10 (2)	< 0.001	74 (12)	20 (3)	< 0.001	110 (9)	30 (3)	< 0.001
*Vibrio*	10 (2)	1 (0.2)	0.006	76 (13)	2 (0.3)	< 0.001	86 (7)	3 (0.3)	< 0.001
Rotavirus	122 (20)	13 (2)	< 0.001	133 (22)	15 (3)	< 0.001	255 (21)	28 (2)	< 0.001
Adenovirus	20 (3)	5 (1)	0.002	15 (3)	1 (0.2)	< 0.001	35 (3)	6 (1)	< 0.001
Norovirus	92/597 (15)	36/599 (6)	< 0.001	58 (10)	46 (8)	NS	150/1,197 (13)	82/1,199 (7)	< 0.001
*Giardia*	32 (5)	90/598 (15)	< 0.001	51 (9)	103 (17)	< 0.001	83 (7)	193/1,198 (16)	< 0.001
*Cryptosporidium*	12 (2)	2 (0.3)	0.007	14 (2)	8 (1)	NS	26 (2)	10 (1)	0.007
*Cyclospora*	1/598 (0.2)	1/597 (0.2)	NS	9/591 (2)	7/594 (1)	NS	10/1,189 (1)	8/1,191 (1)	NS
Multiple pathogens	104/361 (29)	61/264 (23)	0.001	209/469 (45)	113/340 (33)	0.001	313/830 (38)	174/604 (29)	< 0.001
No pathogen identified	239 (40)	336 (56)	< 0.001	131 (22)	260 (43)	< 0.001	370 (31)	596 (50)	< 0.001

EAEC = enteroaggregative *E. coli*; EIEC = enteroinvasive *E. coli*; EPEC = enteropathogenic *E. coli*; ETEC = enterotoxigenic *E. coli*; NS = not significant.

* Two-tailed χ^2^ without continuity correction; two-tailed Fisher’s exact test for comparisons with *n* < 5.

We further characterized the major bacteria and viruses identified. Of 84 *Shigella* isolates from 72 cases and 11 control subjects, *Shigella flexneri* was predominant in 49 of 84 isolates (58%), followed by *Shigella sonnei* in 23 of 84 (28%), *Shigella boydii* in 7 of 84 (8%), and *Shigella dysenteriae* in 5 of 84 (6%). The most common serotype was *S. flexneri* 2a, identified in 24 of 49 isolates (49%), follow by *S. flexneri* 6 identified in 8 of 49 (16%), *S. flexneri *4 and 2b identified in 4 of 49 (8%), and *S. flexneri *3a found in 3 of 49 (6%). Of the available 72 unique ETEC isolates from 65 cases, 38 of 72 (53%) were heat-stable toxin-producing ETEC, 15 of 72 (21%) were heat-labile toxin-producing ETEC, and 19 of 72 (26%) were heat-labile and heat-stable ETEC. Of the available 39 unique ETEC isolates from 38 control subjects, 15 of 39 (38%) were heat-stable toxin-producing ETEC, 19 of 39 (49%) were heat-labile toxin-producing ETEC, and 5 of 39(13%) were heat-labile and heat-stable ETEC. Heat-stable toxin-producing ETEC or heat-labile and heat-stable ETEC were found significantly more in cases than control subjects (52 of 72 [72%] versus 19 of 39 [48%], *P* = 0.02). *Aeromonas *spp. were identified in 110 cases and 30 control subjects, and were further characterized as *Aeromonas caviae *in 45% versus 36%, *Aeromonas hydrophila *in 26% versus 42%, and *Aeromonas veronii *in 28% versus 22% of cases and control subjects, respectively. Data showed *A. caviae* was the predominant *Aeromonas* spp. in the case group. Of 214 total *Campylobacter* isolates, 145 of 214 (68%) were *Campylobacter jejuni*, 50 of 214 (23%) were *Campylobacter coli*, and 19 of 214 (9%) were *Campylobacter *spp. Norovirus was detected in 232 children, including 209 of 232 (90%) that were identified as genogroup GII, 21 of 232 (9%) that were genogroup GI, and 2 of 232 (1%) that were mixed genogroup GI and GII.

Comparing the frequency of etiological agents between the two surveillance sites, a diarrheal pathogen was detected in 80% of cases at Kanti Children’s Hospital compared with 60% at Bharatpur Hospital. Bacterial pathogens were isolated more commonly from stool samples collected at Kanti Children’s Hospital and diarrhea associated with bacterial pathogens. *Shigella*, ETEC, *Aeromonas*, and *Vibrio* were observed more frequently at Kanti Children’s Hospital than at Bharatpur Hospital. The frequency of viral pathogens (rotavirus, adenovirus, astrovirus, and norovirus) were comparable at the two hospitals.

**Figure 1.**
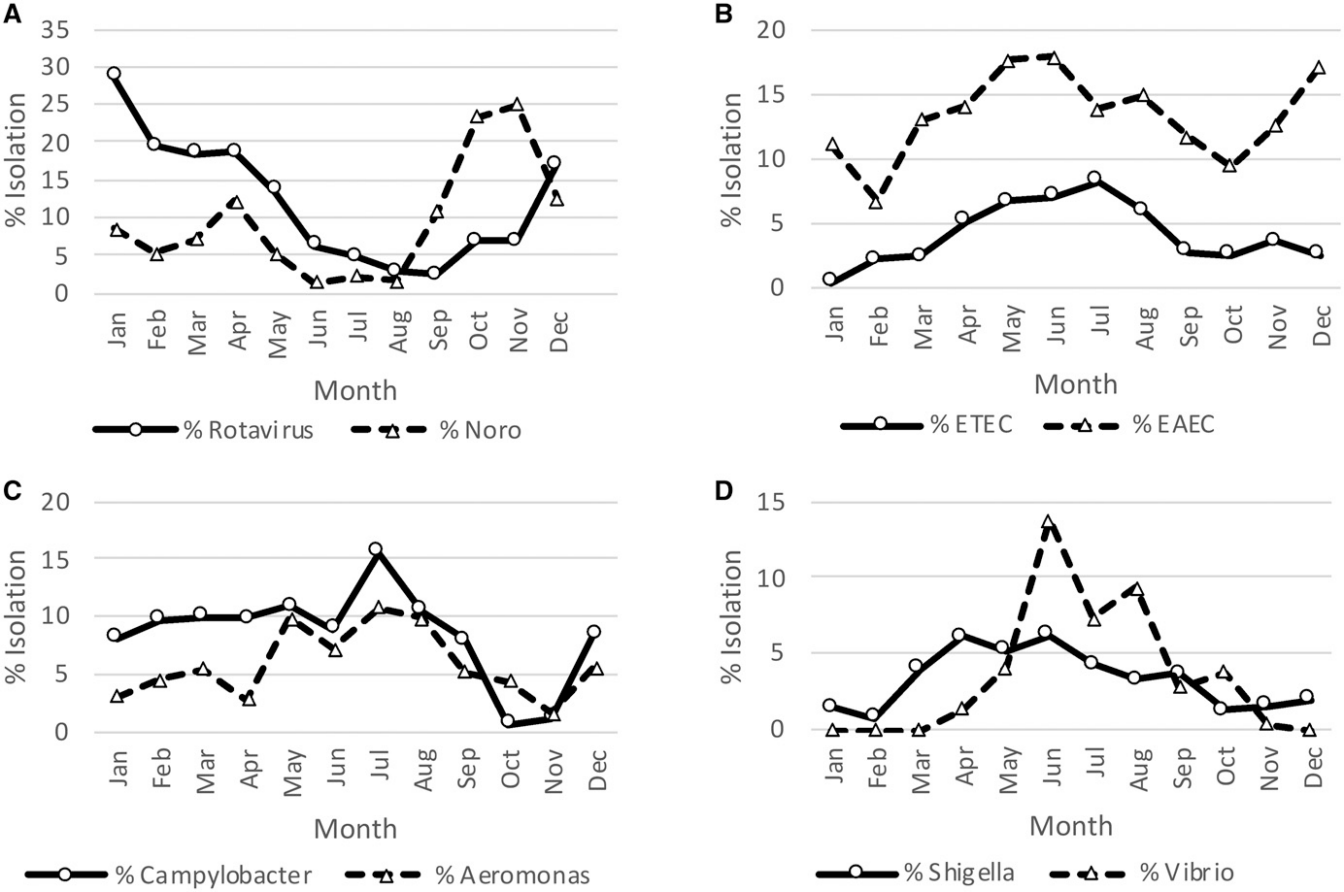
Monthly detection of (**A**) rotavirus and norovirus (Noro), (**B**) enterotoxigenic *Escherichia coli* (ETEC) and enteroaggregative *E. coli*, (**C**) *Campylobacter* and *Aeromonas*, and (**D**) *Shigella* and *Vibrio*

Infections with multiple organisms were common at both Bharatpur Hospital and Kanti Children’s Hospital among both cases and control subjects (29% versus 23% for Bharatpur Hospital and 45% versus 33% for Kanti Children’s Hospital) (Table 2). In general, viral infections (rotavirus and norovirus) and the bacterial pathogen EAEC were more common in children during the first 2 years of life, whereas the bacterial pathogens (*Shigella*, *Campylobacter*, ETEC, and *Vibrio*) were more common in children older than 2 years (Table 3). With regard to seasonality, rotavirus was detected more frequently during the winter and dry season (December–April), whereas norovirus cases were found from September through December (Figure 1). Bacterial infections tended to occur more commonly distributed during the summer and rainy season (May–August), with the exception of EAEC, which was seen all year with a peak in May (Figure 1).

**Table 3:** Numbers and percentages of pathogens identified in stool samples from children by age group in Bharatpur Hospital and Kanti Children’s Hospital

Enteropathogen	Bharatpur Hospital, *n *(%)						Kanti Children’s Hospital, *n *(%)					
	Age 3–12 months		Age 12–24 months		Age 24–60 months		Age 3–12 months		Age 12–24 months		Age 24–60 months	
	Case (*N =* 259)	Control (*N =* 194)	Case (*N =* 207)	Control (*N =* 168)	Case (*N =* 134)	Control (*N =* 238)	Case (*N =* 256)	Control (*N =* 217)	Case (*N =* 167)	Control (*N =* 142)	Case (*N =* 177)	Control (*N =* 241)
*Campylobacter*	5 (2)	8 (4)	4 (2)	10 (6)	2 (1)	11 (5)	31 (12)	18 (8)	24 (14)	25 (18)	31 (18)	35 (15)
*Salmonella*	5 (2)	3 (2)	3 (1)	6 (4)	4 (3)	5 (2)	10 (4)	10 (5)	7 (4)	8 (6)	8 (5)	3 (1)
*Shigella*	0 (0)	0 (0)	7 (3)	0 (0)	17 (13)	1 (0.4)	5 (2)	0 (0)	12 (7)	1 (1)	31 (18)	9 (4)
*Escherichia coli*												
ETEC	5/157 (3)	0/117 (0)	6/120 (5)	4/113 (4)	8/77 (10)	10/176 (6)	15/166 (9)	5/159 (3)	12/124 (10)	7/113 (6)	19/128 (15)	12/192 (6)
EPEC	8/171 (5)	8/131 (6)	7/140 (5)	10/126 (8)	7/90 (8)	18/193 (9)	8/187 (4)	8/178 (4)	15/133 (11)	11/127 (9)	11/141 (8)	28/213 (13)
EAEC	43/171 (25)	32/131 (24)	20/140 (14)	25/126 (20)	7/90 (8)	21/193 (11)	45/187 (24)	47/178 (26)	28/133 (21)	24/127 (19)	13/141 (9)	20/213 (9)
*Aeromonas*	3 (1)	1 (0.5)	8 (4)	5 (3)	25 (19)	4 (2)	25 (10)	6 (3)	13 (8)	4 (3)	36 (20)	10 (4)
*Vibrio*	1 (0.4)	0 (0)	0 (0)	0 (0)	9 (7)	1 (0.4)	13 (5)	1 (0.5)	10 (6)	0 (0)	53 (30)	1 (0)
Rotavirus	52 (20)	3 (2)	54 (26)	5 (3)	16 (12)	5 (2)	69 (27)	7 (3)	48 (29)	1 (1)	16 (9)	7 (3)
Adenovirus	13 (5)	1 (0.5)	4 (2)	1 (0.6)	3 (2)	3 (1)	5 (2)	0 (0)	7 (4)	0 (0)	3 (2)	1 (0.4)
Norovirus	32/258 (12)	16/193 (8)	53/205 (26)	15 (9)	7 (5)	5 (2)	31 (12)	22 (10)	23 (14)	13 (9)	4 (2)	11 (5)
*Giardia*	4 (2)	8 (4)	10 (5)	22/167 (13)	18 (13)	60/237 (25)	4 (2)	6 (3)	14 (8)	20 (14)	33 (23)	76 (31)
*Cryptosporidium*	5 (2)	0 (0)	4 (2)	2 (1)	3 (2)	0 (0)	6 (2)	2 (1)	2 (1)	1 (1)	6 (3)	5 (2)
No pathogens Identified	123 (47)	132 (68)	68 (33)	84 (50)	48 (36)	120 (50)	81 (32)	114 (53)	26 (16)	59 (42)	24 (14)	87 (36)

EAEC = enteroaggregative *E. coli*; EPEC = enteropathogenic *E. coli*; ETEC = enterotoxigenic *E. coli*.

Viral infections, rotavirus, norovirus, and adenovirus tend to present with watery stool without blood (56%–88%) and with vomiting (83%–89%) (Table 4). *Shigella* infections were associated with fever (76%) and bloody diarrhea (31%). Clinical presentations of infections by *Vibrio* spp. were similar to viral gastroenteritis, with watery diarrhea without blood (62%) and with vomiting (81%) (Table 4).

**Table 4:** Clinical signs and symptoms in diarrhea cases with stool samples with a single enteropathogen identified

Enteropathogen	Patients, *n*	Patients with signs or symptoms, *n* (%)			
		Watery stool	Bloody stool	Fever	Vomiting
*Campylobacter*	24	7 (29)	1 (4)	10 (42)	15 (63)
*Salmonella*	11	4 (36)	0 (0)	8 (73)	4 (36)
*Shigella*	35	13 (37)	11 (31)	26 (74)	20 (57)
ETEC	14	4 (29)	0 (0)	7 (50)	9 (64)
*Aeromonas*	23	13 (57)	1 (4)	8 (35)	16 (70)
*Vibrio*	21	13 (62)	0 (0)	8 (38)	17 (81)
Rotavirus	161	103 (64)	0 (0)	57 (35)	143 (89)
Adenovirus	16	14 (88)	0 (0)	9 (56)	14 (88)
Norovirus	95	53 (56)	0 (0)	23 (24)	79 (83)

ETEC = enterotoxigenic *Escherichia coli*.

Antibiotic resistance for selected bacterial pathogens isolated from both cases and control subjects is shown in Table 5. Azithromycin was effective against most bacterial pathogens. Ciprofloxacin had activity against most bacteria except *Campylobacter*,* S. dysenteriae*,* S. flexneri*, and EAEC, whereas resistance to nalidixic acid was present in more than 40% of isolates for all bacteria except enteroinvasive *E. coli*. Bacterial isolates were resistant to nalidixic acid, ampicillin, sulfonamide, and tetracycline when tested against these antibiotics.

**Table 5:** Antimicrobial resistance of bacterial isolates from stool samples from children age 3 months to 5 years with and without diarrhea in Bharatpur and Kathmandu, Nepal

Enteropathogen	Isolates tested, *n*	Isolates with antibiotic resistance, *n *(%)							
		Macrolide	Quinolone		Penicillin		Sulfonamide	Tetracycline	Cephalosporin
		AZM	NA	CIP	AM	AMC	SXT	TE	CFM
*Campylobacter*									
* C. jejuni*	137	0 (0)	99 (72)	96 (70)	5 (4)	0 (0)	81 (59)	41 (30)	93/110 (85)
* C. coli*	53	1 (2)	39 (74)	38 (72)	1 (2)	0 (0)	31 (58)	21 (40)	41/42 (98)
* Campylobacter* spp.	19	0 (0)	8 (42)	7 (37)	1 (5)	0 (0)	8 (42)	0 (0)	7/17 (41)
* Salmonella*	73	0 (0)	32 (44)	0 (0)	24 (33)	ND	16 (22)	1 (1)	22/56 (39)
*Shigella*									
* S. boydii*	7	0 (0)	3 (43)	0 (0)	2 (29)	0 (0)	3 (43)	2 (29)	1/5 (20)
* S. dysenteriae*	5	0 (0)	3 (60)	2 (40)	3 (60)	1 (20)	5 (100)	4 (80)	0/4 (0)
* S. flexneri*	49	0 (0)	31 (63)	11 (22)	27 (55)	7 (14)	33 (67)	38 (78)	0/35 (0)
* S. sonnei*	23	0 (0)	22 (96)	1 (4)	0 (0)	0 (0)	19 (83)	19 (83)	0/18 (0)
*Escherichia coli*									
* *ETEC	103	1 (1)	53 (51)	2 (2)	48 (47)	ND	41 (40)	22 (21)	8/36 (3)
* *EPEC	139	20 (14)	56 (40)	1 (1)	59 (42)	ND	49 (35)	36 (26)	27/125 (22)
* *EAEC	330	36 (11)	277 (84)	126 (38)	288 (87)	ND	220 (67)	167 (51)	173/288 (60)
* *EIEC	8	0 (0)	0 (0)	0 (0)	3 (37)	ND	2 (25)	2 (25)	1 (12)
*Aeromonas*	160	7 (4)	61 (38)	6 (4)	159 (99)	ND	11 (7)	24 (15)	8/101 (8)
*Vibrio*	95	0 (0)	95 (100)	0 (0)	1 (1)	ND	88 (93)	88 (93)	0 (0)

AM = ampicillin; AMC = amoxicillin/clavulanic acid; AZM = azithromycin; CFM = cefixime; CIP = ciprofloxacin; EAEC = enteroaggregative *E. coli*; EIEC = enteroinvasive *E. coli*; EPEC = enteropathogenic *E. coli*; ETEC = enterotoxigenic *E. coli*; NA = nalidixic acid; ND = not done; SXT = trimethoprim/sulfamethoxazole; TE = tetracycline.

## DISCUSSION

Acute gastroenteritis is a major public health problem in developing countries, especially among young children. Determination of etiological agents causing diarrhea in children younger than 5 years is difficult, with detection of enteric pathogens even in children without diarrhea. In our study we applied microbiologic methods to detect a diverse range of enteric pathogens in both diarrheal and non-diarrheal stools. Rotavirus, *Shigella*, and ETEC have been reported extensively as important causes of childhood diarrhea.^2^^,^^6^ Our findings support this and we also identified other enteric pathogens. Adenovirus, norovirus, *Vibrio*, and *Aeromonas* are associated significantly with acute diarrheal disease in children younger than 5 years in Nepal. Various distributions of enteric pathogens in different age groups were noted in our study, with greater numbers of viral infections seen during the first 2 years of life and bacterial infections noted in later childhood.

Rotavirus is reported as a leading cause of viral gastroenteritis; its reported frequency in diarrhea in Nepal ranges from 15% to 45%.^15^^–^^18^ This finding agrees with ours and suggests the potential importance of rotavirus vaccination for children in Nepal to reduce this significant public health burden. Norovirus, reported as the second leading pathogen associated with viral gastroenteritis,^19^^,^^20^ has been less investigated in Nepal. In our study we documented that norovirus was associated significantly with diarrhea, in contrast to a previous case–control study^21^ of infantile diarrhea in Nepal that reported norovirus in a similar proportion in both cases and control subjects. The frequency of norovirus genotype GII infections in our study was estimated at 74 of 372 (20%) in the 1- to 2-year age group and 58 of 514 (11%) in the age group younger than 1 year. This is similar to the findings of the community-based multi-site birth cohort, malnutrition and enteric disease (MAL-ED) study,^22^ which reported a significant burden of diarrhea associated with norovirus genotype GII infection in children in their second year of life. Likewise, Hoa-Tran et al.^23^ also reported norovirus in 8% of Nepalese children younger than 5 years , predominantly in hospitalized children age 6 to 23 months between 2005 and 2011. A better understanding of norovirus epidemiology is essential for intervention planning with appropriate vaccine development, good clinical trials, and implementation.

*Cyclospora* is a protozoal pathogen causing prolonged diarrhea in mainly immune-compromised hosts, and expatriate and traveler populations in developing countries.^24^^,^^25^ A study conducted in Nepalese children younger than 5 years in the Kathmandu area during peak *Cyclospora* season reported a 12% prevalence of *Cyclospora* infections among children with diarrhea, in contrast to our findings, which noted that *Cyclospora* was detected overall in only 1% to 2% of children.^26^ However, further studies in indigenous populations are needed to understand more fully the importance and epidemiology of *Cyclospora*.

Asymptomatic carriage of enteric organisms such as non-typhoidal *Salmonella*, *Giardia*, and *Campylobacter* has been reported in several studies in developing countries.^9^^,^^27^ The data obtained in our study support those findings. *Giardia lamblia* was detected in a significantly greater proportion in control subjects than in cases. This has also been observed in case–control studies in Bangladesh^25^ and in a rural area of Thailand,^9^ and as part of the Global Enteric Multicenter study,^7^ with no clear explanation. Although *Campylobacter* contributed to the greatest burden of diarrhea during the first year of life at the MAL-ED community-based study sites in Brazil, Peru, and South Africa, it was identified in Nepalese children with diarrhea mostly after age 12 to 24 months,^28^ and has been associated with dysentery.^6^ In our study, *Campylobacter* was detected, but not associated significantly with diarrhea. However, we observed a greater number and frequency of isolates with increasing childhood age.

Easy access to antibiotics from pharmacies without a prescription and the lack of an antibiotic stewardship policy contribute to unnecessary administration of antibiotics for the treatment of diarrheal disease in many parts of the world. This can be problematic, both in terms of economic burden related to cost of medications and health care, and it also contributes to antibiotic resistance. Therefore, it is important to promote oral rehydration therapy and to administer zinc to reduce prolonged, persistent diarrhea^29^ instead of using unnecessary medication for management of acute diarrhea in children. In our study, *Shigella* isolates were frequently resistant to NA and trimethoprim–sulfamethoxazole, the antibiotics that were previously commonly used for the treatment of diarrheal disease. This finding is similar to what was reported from rural Thailand,^9^ where third-generation cephalosporins and azithromycin were found to be the most susceptible antibiotics. Similar findings were reported by Dhital et al. in 2007.^30^ A high prevalence of nalidixic acid and fluoroquinolone-resistant *Campylobacter* has been well documented in studies from Nepal^31^ and Thailand.^32^ Azithromycin has been recommended as an alternative antimicrobial regimen for the treatment of multidrug-resistant and quinolone-resistant *Salmonella typhi* and other *Salmonella* infections.^33^ No azithromycin-resistant *Salmonella* and *Shigella* isolates were noted in our study, but have been reported in other places such as Bangladesh.^34^^,^^35^ The emergence of antimicrobial resistance and multidrug-resistant bacteria are of the greatest global concern for the treatment of infections. Third-generation cephalosporins are being used increasingly for the treatment of diarrheal disease,^30^ which can lead to increased prevalence of extended-spectrum beta lactamase bacteria, creating challenges in the treatment of more serious diseases. Therefore, it is important to ensure the appropriate use of antibiotics and establish a systematic monitoring network for antibiotic resistance.

In our study, inclusion of a control group enabled us to associate causality and asymptomatic carriage of each viral, bacterial, and parasitic organism studied, unlike other hospital-based diarrheal studies conducted in Nepal, in which only cases with acute gastroenteritis were included, with focused testing for specific pathogens. Moreover, the application of microbiologic culture techniques allowed us to obtain bacterial isolates for antimicrobial susceptibility testing to address antimicrobial resistance issues. The bacterial archives can be further studied on resistance mechanisms with sequencing for bacterial evolution in the future. Although not available at the time of the study, current molecular panels would increase the detection frequency for several of the pathogens. Despite of a limitation of not including more associated risk factors and unmatched children age between cases and control subjects, our study has good strength in having included severe cases from hospital inpatients to study the etiology of diarrhea in these children.

Our study documents rotavirus as an important etiology in acute diarrhea among children younger than 5 years in Nepal, followed by norovirus and the bacteria *Shigella*, ETEC, *Vibrio cholera*, and *Aeromonas*. Identifying pathogen burden in combination with relevant clinical and epidemiological information is important for development of appropriate public health interventions for the prevention and treatment of diarrheal disease among children in Nepal.
